# WaterSpy: A High Sensitivity, Portable Photonic Device for Pervasive Water Quality Analysis

**DOI:** 10.3390/s19010033

**Published:** 2018-12-21

**Authors:** Nikolaos Doulamis, Athanasios Voulodimos, Anastasios Doulamis, Matthaios Bimpas, Aikaterini Angeli, Nikolaos Bakalos, Alessandro Giusti, Panayiotis Philimis, Antonio Varriale, Alessio Ausili, Sabato D’Auria, George Lampropoulos, Matthias Baer, Bernhard Schmauss, Stephan Freitag, Bernhard Lendl, Krzysztof Młynarczyk, Aleksandra Sosna-Głębska, Artur Trajnerowicz, Jarosław Pawluczyk, Mateusz Żbik, Jacek Kułakowski, Panagiotis Georgiadis, Stéphane Blaser, Nicola Bazzurro

**Affiliations:** 1 National Technical University of Athens (NTUA), 15773 Athens, Greece; thanosv@mail.ntua.gr (A.V.); adoulam@cs.ntua.gr (A.D.); mbibas@esd.ece.ntua.gr (M.B.); katerina.angeli.doulami@gmail.com (A.A.); bakalosnik@mail.ntua.gr (N.B.); 2 University of West Attica, 12243 Athens, Greece; 3 Cyprus Research and Innovation Center Ltd. (CyRIC), 2414 Nicosia, Cyprus; alessandro@cyric.eu (A.G.); p.philimis@cyric.eu (P.P.); 4 Consiglio Nazionale delle Ricerche (CNR), 80131 Naples, Italy; antonio.varriale@isa.cnr.it (A.V.); alessio.ausili@isa.cnr.it (A.A.); s.dauria@ibp.cnr.it (S.D.A.); 5 A.U.G. Signals Hellas (AUG), 10674 Athens, Greece; lampro@augsignals.com; 6 Friedrich-Alexander-Universität Erlangen-Nürnberg (FAU), 91058 Erlangen, Germany; matthias.baer@fau.de (M.B.); bernhard.schmauss@fau.de (B.S.); 7 Technische Universität Wien (TUW), 1060 Vienna, Austria; stephan.freitag@tuwien.ac.at (S.F.); bernhard.lendl@tuwien.ac.at (B.L.); 8 VIGO Systems SA (VIGO), 05-850 Ożarów Mazowiecki, Poland; kmlynarczyk@vigo.com.pl (K.M.); asosna@vigo.com.pl (A.S.-G.); atrajnerowicz@vigo.com.pl (A.T.); jpawluczyk@vigo.com.pl (J.P.); mzbik@vigo.com.pl (M.Ż.); jkulakowski@vigo.com.pl (J.K.); 9 Alpes Lasers SA (ALPES), 2072 St Blaise, Switzerland; panagiotis.georgiadis@alpeslasers.ch (P.G.); stephane.blaser@alpeslasers.ch (S.B.); 10 IREN Gruppo S.p.A. (IREN), 16138 Genoa, Italy; Nicola.Bazzurro@iride-acquagas.it

**Keywords:** water quality monitoring, photonics, Quantum Cascade Lasers, photodetectors

## Abstract

In this paper, we present WaterSpy, a project developing an innovative, compact, cost-effective photonic device for pervasive water quality sensing, operating in the mid-IR spectral range. The approach combines the use of advanced Quantum Cascade Lasers (QCLs) employing the Vernier effect, used as light source, with novel, fibre-coupled, fast and sensitive Higher Operation Temperature (HOT) photodetectors, used as sensors. These will be complemented by optimised laser driving and detector electronics, laser modulation and signal conditioning technologies. The paper presents the WaterSpy concept, the requirements elicited, the preliminary architecture design of the device, the use cases in which it will be validated, while highlighting the innovative technologies that contribute to the advancement of the current state of the art.

## 1. Introduction

The indubitable vitality of freshwater as a necessary resource for all facets of human life and activity is a self-proving argument of the severe threat posed by the pollution and waste of freshwater. The water distribution system is susceptible in accidental or intentional contamination, due to the distributed nature of their geography and access points [1]. Hazardous contaminants can be introduced into the system by intentional sabotage, illegal disposal of wastewater, terrorist attack, accident or due to naturally occurring incidences (such as negative pressure siphoning contaminated water into broken pipes, network backwash, erosion of chromium rich soil, etc.). The decline in water quality can be put down to a variety of causes including: long-range atmospheric transport of pollutants, discharge of toxic chemicals, over-pumping of aquifers, and contamination with substances that promote algal growth [2].

Each water body, storage tank or water network segment can contain dramatically different contaminant concentration levels. Water quality monitoring is therefore a fundamental tool in the management of freshwater resources, public safety and the economy. Monitoring is defined by the International Organization for Standardization (ISO) as: “the programmed process of sampling, measurement and subsequent recording or signaling, or both, of various water characteristics, often with the aim of assessing conformity to specified objectives” [3]. Pervasive and on-line water quality monitoring data is therefore critical to detecting environmental pollution and reacting in the best possible way, in order to avoid human health hazards [4].

In the past, the lack of methods of sufficient sensitivity, speed, cost-effectiveness and ability to detect and measure indigenous microbial cells and related pathogens prevented the research community from profoundly comprehending the indigenous bacterial flora, its characteristics and the relevant processes taking place in water treatment and distribution systems. For many years, routine monitoring and evaluation of hygiene revolved mainly around the detection of indicators for faecal pollution using plating methods, as well as the detection of cultivable heterotrophic microbes as a measure of the general microbiological quality of water [4]. European legislation currently dictates the measurement of only three microbiological parameters, namely heterotrophic plate counts (HPC) and the two bacterial indicators *Escherichia coli* and *Enterococcus* spp.

Cultivation-based heterotrophic plate counts (HPC) are universally employed as a general microbial quality factor in water treatment and distribution systems, as is the case in [5,6], even though there are many more microbial cells in drinking water in comparison to the numbers that can be cultured on synthetic growth media. Total bacterial cell concentrations (determined for example with microscopy) are normally not considered during drinking water treatment as either design, operative or legislative parameter. A proper understanding of microbial survival and growth during drinking water treatment and distribution starts with the ability to quantify all the microorganisms accurately and rapidly. Some 90–99%, or even more, of the bacterial cells detected in aqueous and terrestrial environments cannot be cultivated in the laboratory with the methods presently used. This is due to the wide variety of contaminant strains, co-cultures and the fact that some bacteria appear to grow only when inoculated above a certain density [7]. This huge discrepancy between cultivable and total cell counts has been known for a considerable time and is commonly referred to as “the great plate count anomaly”. This raises the question of the viability and activity of the fraction of non-cultivable bacterial cells.

**Figure 1:**
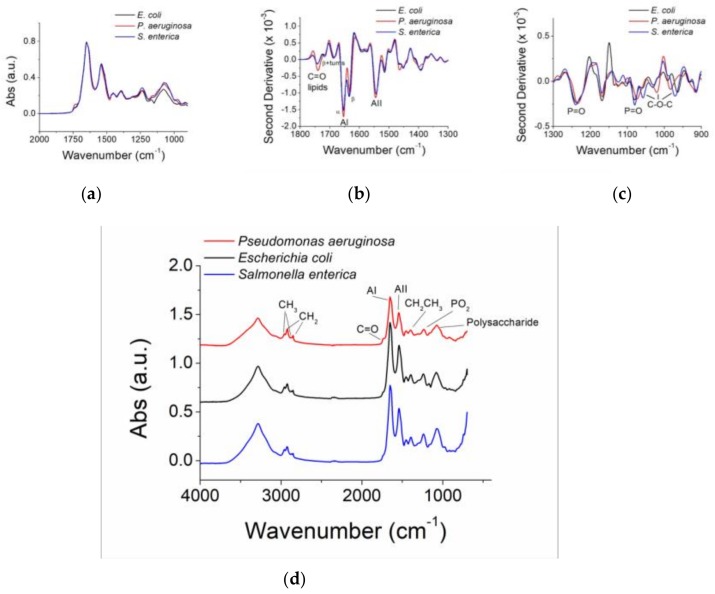
Fingerprint IR band of the selected analytes with respect to wavenumber: (**a**) & (**d**): absolute, and (**b**) & (**c**): second derivative.

The WaterSpy project [8] aims at answering to the aforementioned limitations through the development of a novel, compact, cost-effective photonic device, operating in the spectral range of 6–10 μm and suitable for pervasive water quality sensing. This is compatible with the fingerprint IR of the three analytes (i.e., *Pseudomonas aeruginosa*, *Escherichia coli*, *Salmonella enterica*), as displayed in Figure 1. In this paper, we present the WaterSpy approach, which is based on the combined use of advanced Quantum Cascade Lasers (QCLs) employing the Vernier effect, used as light source, and novel, fibre-coupled, fast and sensitive Higher Operation Temperature (HOT) photodetectors, used as sensors. Together with these new components, optimised laser driving and detector electronics as well as laser modulation and signal conditioning and signal processing concepts will be developed. Attenuated total reflectance (ATR) spectroscopy will be used to get advantage of the phenomenon of total internal reflection, and give rise to the biochemical profile of the surface chemistry of the sample. Targeted analytes will be specific heterotrophic bacterial cells. Several novel and smart techniques are employed in order to increase Signal-to-Noise Ratio (SNR) and get an accurate reading, including antibodies capable of binding the targeted analytes and a novel sample pre-concentration method. WaterSpy technology will be integrated, for validation and testing purposes, to a commercially successful water quality monitoring platform (TRITON by AUG), in the form of a portable device add-on, thus minimizing system integration effort. WaterSpy will be used in the field for water quality analysis of large water bodies and critical points of water distribution networks, providing pervasive quality monitoring. This will be demonstrated in two different demo sites in Italy, one in the urban water distribution network and one in the aqueducts of a water treatment plant.

The remainder of this paper is structured as follows: Section 2 presents a survey of the state of the art in a series of topics relevant to WaterSpy and highlights the novel approaches of WaterSpy. Section 3 presents the results of the requirements elicitation process, while Section 4 provides a description of the main use cases of WaterSpy. A brief description of the design and architecture of WaterSpy is given in Section 5, while Section 6 presents and discusses some initial experimental validation results of the proposed implementation. Finally, Section 7 concludes the paper.

## 2. Survey of Existing Related Technologies and WaterSpy’s Innovation

The WaterSpy project advances the state of the art in a series of topics; therefore, in this section a brief survey of the state of the art for each topic is provided, along with the innovation introduced by WaterSpy.

### 2.1. Quantum Cascade Lasers (QCLs)

Quantum cascade lasers (QCLs) were introduced in 1994 and are a type of semiconductor laser which use epitaxially grown quantum wells containing lasing state electrons. QCLs are based on a different physical principle compared to diode lasers. In a diode laser, transitions happen between the conduction band and valence band of the semiconductor material. On the contrary, the lasing transition in a QCL occurs between states within a given quantum well. This approach has the benefit that the electron responsible for the emission of the photon tunnels into the next quantum well; therefore, multiple photons can be generated by a single electron, thereby making QCLs very efficient. The term “quantum cascade” has been derived from the tunneling of the electrons from one well to the next one. In addition, the wavelength of the lasing transition depends from the physical structure of the device, since the well depths can be engineered during the fabrication process by controlling layer depths. In this context, a careful design of the layers can result to achieving lasing at wavelengths as short as 2.75 µm [9] and as long as 161 µm (1.9 THz). The longer wavelength devices require cryogenic cooling still, but room temperature operation has been observed to at least 16 µm [10]. Interest is mainly concentrated in the mid-infrared (3.5–13 µm) and the Terahertz spectrum (2–5 THz ≈ 60–150 µm). Twenty years after their discovery, QCLs are certainly the most mature and used laser source in the Mid-IR spectral region [11]. Laser operation between 3–20 µm has been proven, and high power and continuous wave operation has been observed especially in the 4 to 10 µm spectral range. Laser efficiencies up to 27% have been proven [12].

Vernier lasers have also been fabricated by numerous groups using QCL material [13]. Nevertheless, in all cases, the devices composed of multi-section QCL where the laser optical cavity was separated in different places in order to ensure independent electrical pumping of the mirrors and the gain section. The tuning of the mirror’s reflectivities was then obtained modulating the current across the laser structure, modulating therefore at the same time also the laser gain and resulting in spectral instabilities. Also, partial reflection of photons at the section separations can occur, increasing possible mode-jumps and unpredictable spectral behavior of the laser system.

The WaterSpy device will incorporate a novel micro-heater, developed by ALPES, that allows for a compact and fast modulation of the mirror’s temperature without need for modulating the current through the mirror, while also reducing the risk for partial reflections [14]. This heater has proven to increase largely the stability of the laser emission in Vernier QCLs. WaterSpy will be the first project to result in a compact system which uses a large number of sources in the Mid-IR.

### 2.2. Higher Operation Temperature (HOT) Photodetectors

The first uncooled photoconductive and photoelectromagnetic LWIR photodetectors used for detection of CO_2_ laser radiation were demonstrated in 1972 by VIGO Systems, one of the partners of the WaterSpy project. The devices were based on HgCdTe variable gap heterostructures grown using isothermal vapour phase deposition [15]. The devices have been steadily improved by the group and eventually commercialized by VIGO. The devices were optimized for operation at any wavelength within the 2 to 16 µm spectral range and at ambient temperature or temperatures achievable with Peltier coolers (190 to >300 K).

Various techniques have been used to improve performance of the devices without cryogenic cooling, such as suitable selection of the composition and doping profiles of the heterostructures, enhanced absorption with multiple pass of IR radiation, use of monolithic optical immersion to reduce physical size of the detector, cascade design with multiple photovoltaic cells connected in series and other solutions. The selection of these parameters was achieved in a heuristic/experimental way.

In WaterSpy, an important goal will be to develop fibre-coupled, fast and sensitive, HOT photodetectors with spectral response extended to longer wavelength, operating at ambient temperature or cooled in a cost-efficient and convenient way, using Peltier coolers. This goal will be achieved, based on previous research to reduce cooling requirements and additional modifications of the HgCdTe heterostructure to obtain high speed and low dark current. The GaAs immersion lens that will be used in the HOT photodetector, increases apparent optical area of the photodetector by more than 2 orders of magnitude in comparison with the non-immersed device of the same physical area. This results in a corresponding reduction of the dark current & capacitance, which is essential for the performance of the device at high frequencies. In this context, WaterSpy will design and manufacture a prototype fibre-coupler for the LWIR detector and investigate its properties. The developed device will enable detection of the IR radiation from 6 to 10 μm via optical fibre.

### 2.3. Photonics-Based Detection of Water Pollutants

Over the last fifty years, sensitive and selective analytical tools based on spectroscopic and chromatographic technologies have been designed and created for monitoring a plethora of chemicals compounds and bacteria strains, as in [16,17,18]. Normally the analysis process involves periodic water sample collection from the field by appropriately trained experts, followed by the transfer of the samples to the laboratory for analysis. This approach gives inadequate results in terms of both spatial and temporal resolution. Several efforts have been made ([19,20]) in order to overcome this problem and develop sensors and biosensors for monitoring the levels of organic, inorganic and microbiological pollutants in aquatic environments, without the need of continuous visits to the lab and prolonged analysis times.

Mid-infrared (MIR) sensors based on attenuated total reflectance (ATR) have been developed to detect chemicals, such as BTX compounds and microbiological pollutants in water and in food. Since its development, Infrared (IR) spectroscopy has been extensively used to provide structural composition of chemical compounds, and has become a useful tool for the detection of a number of environmental relevant pollutants [21]. Fourier transform infrared (FT-IR) absorbance spectroscopy (in the mid-IR range, often defined as 4000–400 cm^−1^ or 2.5–25 μm [22]) has sufficient resolution to distinguish inorganic and organic compounds and intact microbial cells at the strain level. Attenuated total reflectance (ATR) is the common IR technique used for detection chemical compound in solution. Common IR sampling techniques used for microbial characterization are transmittance, diffuse reflectance, attenuated total reflectance (ATR), and micro-spectroscopy. The complex FT-IR spectra of bacteria reflect the total biochemical composition of the microorganism, with bands owing to major cellular constituents including proteins, lipids, nucleic acids, polysaccharides, and phosphate-carrying compounds. ATR-FTIR spectroscopy has been used before to detect E.coli and P. aeruginosa successfully [9].

In the last years, different efforts have been made in order to develop multicomponent arrays based on specific biomolecules for label-free sensing application as in [23]. In particular, label-free detection is essential for monitoring of photogenic bacteria in food and water sample exposed to contamination. In this context, the most widely used label-free technique is the surface plasmon resonance (SPR) biosensor. On the other hand, there is the opportunity to exploit the molecular selectivity of mid-IR sensors. Waveguides and fibre-optic materials which are transparent in the mid-IR spectral region allow access to fundamental vibrational fingerprint absorptions of organic molecules [24].

In WaterSpy, specific heterotrophic bacterial cells are targeted: *E. coli*, *P. aeruginosa*, *Salmonella*. Single IR bands of the selected bacteria will be detected, covering proteins, lipids and fatty acids, DNA backbone and RNA. This will allow high specificity of the WaterSpy device. The proposed technique uses the advanced, tuneable QCLs in an ATR configuration, in combination with novel subsystems, such as a smart, antibodies covered surface and an ultrasound pre-concentration method.

### 2.4. The WaterSpy Innovation

Four different commercial products have been identified as relevant to the objectives of the WaterSpy device, but it can be noticed that none of them work in the mid-IR region, as the WaterSpy device is aimed at. Specifically, the tools identified use Hybrid Multispectral Analysis [25], spectral monitoring of fluorescent agents that are created through enzyme activity [26,27], or Enzymatic reaction [28]. Overall, a comparison of WaterSpy with the state of the art can lead to a summary of the innovation of the WaterSpy approach:

● The WaterSpy device will use Quantum Cascade Lasers employing the Vernier effect, in order to completely avoid the use of optics, prisms etc. as in the traditional FTIR approach.
● No reagents are used in the system. This minimizes the use of chemicals and simplifies the transfer of the approach in a compact, portable device with high market penetration potentials.
● The QCLs proposed by WaterSpy allow for a broad tuning of the mirror reflectivity, modulating the laser temperature, while keeping the electrical pumping across the gain medium constant. Additionally, the laser cavity is not separated and therefore the risk for partial reflections is strongly reduced.
● Fibre-coupled, fast and sensitive, HOT photodetectors with spectral response extended to longer wavelength will be developed and used. The GaAs immersion lens that will be used in the HOT photodetector, increases apparent optical area of the photodetector by more than two orders of magnitude in comparison with the non-immersed device of the same physical area.
● The developed photodetector will enable detection of the IR radiation ranging from 6 to 10 μm via optical fibre, terminated by a standard SMA connector.
● WaterSpy will for the first time result in a compact system which uses an increased number of laser sources. The know-how developed in this project will also be an enabling technology for a large number of applications in bio-sensing, security and high speed communications.
● The spectral region targeted presents high importance for several analytes in water. The technology to be developed will be applicable (after some necessary modifications mainly in the QCLs part) for detecting also analytes other than bacteria.

## 3. Requirements Elicitation

In order to elicit requirements for the WaterSpy device, a two-stage process was put forward: an internal and an external survey of key users and stakeholders of water quality control. An analysis of the results of the surveys in conjunction with a review of the characteristics of existing solutions led to a basic set of requirements [29] that drives the design and developments in the WaterSpy project. A brief summary of the outcomes of the surveys is provided in this section, while the full details and results can be found in [29]. The survey population consisted of people with relevant expertise (chemical engineers, electrical engineers, water quality managers, photonics experts), that were both part of the consortium (internal elicitation of requirements) as well as external experts. Out of them, 62% was familiar and has previously used water quality monitoring devices. Moreover, relevant European Commission regulations [30] were taken into account, to ensure that the device’s outcomes adhere to the current legislative framework. These efforts resulted in the following compilation of requirements:

*Requirement #1: Portability*. The developed device should be portable, although not necessarily handheld. Size and weight restrictions are thus implicitly defined, in the sense that they are bound by the necessity to transferable in a vehicle.

*Requirement #2: Suitability to analyze mainly organic pollutants*. Organic pollutants appear to be the focal point of interest of end users in terms of detection target. Organic pollutants include different types of bacteria and especially enterococci bacteria and *E. coli* (*Escherichia coli*). Other types of detections of interest to users include virus detections, fertilizers and pesticides.

*Requirement #3: Reliability—high detection accuracy*. The need for a highly accurate device far outweighs the requirement for computational efficiency, as the surveys showed. Of course, the analysis speed should remain at reasonable levels as well. Time intervals of half day are acceptable, when state of the art techniques for organic pollution detection tend to require approximately a day for culture and final detection. Moreover, current regulations for certifying water for human consumption require the sensitivity of a single specific bacterium per 100 mL of water. This target sensitivity drives the overall accuracy requirements of the device.

*Requirement #4: Cost is more significant than automation*. When it comes to the trade-off between the device’s cost and the level of automation in its functionality, it seems that keeping the cost as low as possible is more important than having additional automatic capabilities. Therefore, the configuration of some parameters of the device can be manually set and controlled, if automating them would significantly raise the cost.

*Requirement #5: Modularity and extensibility*. The design of the device should be modular so as to permit the addition of supplementary plug–ins at later stages, e.g., to allow for additional types of pollutants. A modular and configurable design will also allow for a wider exploitation of the device in the context of different application domains and user types.

*Requirement #6: Networking*. It is necessary that the developed device supports networking so as to be controllable remotely by end-users, mainly for cases of hazardous natural conditions and inaccessible regions. Comparing between the different networking capabilities, WiFi appears to be the most significant one for users.

*Requirement #7: Simple configuration and maintenance*. The WaterSpy device design should permit simple configuration and maintenance procedures, so that the majority of relevant actions can be carried out by the end-users themselves.

*Requirement #8: Signal processing and data analysis capabilities for enhanced performance*. State of the art signal processing and data analysis techniques should be exploited for increased detection accuracy and noise removal. Furthermore, the device should be able to store additional metadata information such as environmental data potentially useful for end-users to better interpret the analysis results. Data encryption policies are also welcome as additional add-ons.

*Requirement #9: Daily operation*. The ability to capture many samples per day is also a requirement. Daily operation is necessary to ensure appropriate monitoring of water quality.

*Requirement #10: Compliance with existing standards*. The device should be compliant with existing standards, as this contributes to better exploitation and adoption, e.g., Water Quality Sampling ISO standard [3,4].

## 4. Waterspy Use Case Description

**Figure 2:**
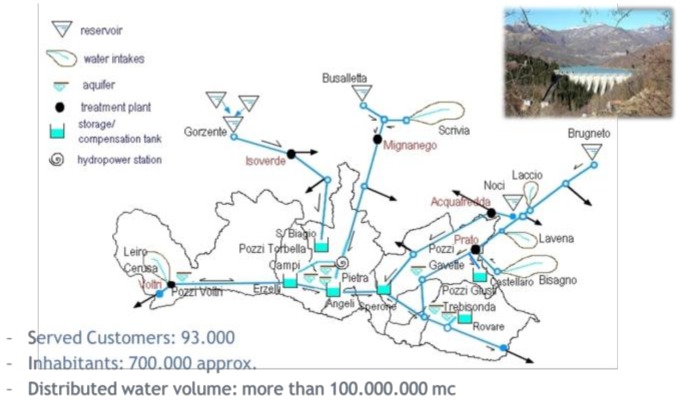
A map of the area around Prato where the WaterSpy device will be installed.

The WaterSpy device will be installed by the drinking water portabilization plant located in Prato (Italy) operated by IRETI and included into the hydraulic infrastructures of the water supply system serving the town of Genoa in Italy (see Figure 2).

The facility is characterized by an average flowrate of about 1300 L/s (dm^3^/s); the treatment train includes several processes which can be summarized in: pre-disinfection with sodium hypochlorite, coagulation/flocculation with aluminum polychloride, filtration through sand, disinfection again with sodium hypochlorite; treated water is supplied to about 198,000 inhabitants living in the city of Genoa.

**Figure 3:**
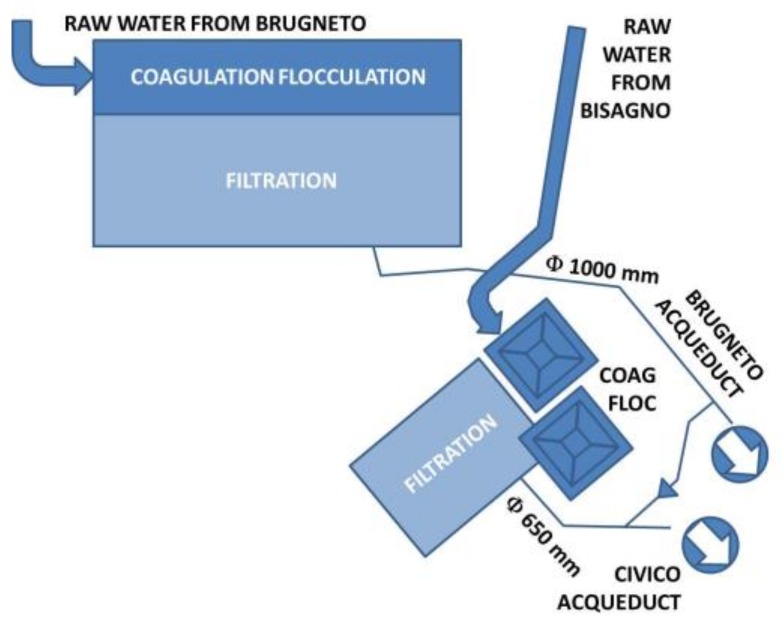
Schematic diagram of the water flow.

At the site where the plant is located raw water from two different supplying sources are treated: from the Brugneto artificial basin (25 Mm^3^.) supplying the homonymous aqueducts and from the water intake on the stream Bisagno supplying the Civico Acqueducts (Figure 3).

The Prato drinking water treatment plant is the terminal part of the Brugneto supply system and consists of 4 open-air sedimentation units, a series of 12 indoor tanks for rapid sand filtration and a chlorine dioxide disinfection facility. The plant has an average flowrate of about 78,000 L (dm^3^) per minute (29.7 MGD).

The exploited water resources, mainly consisting of surface water, show significant variations in turbidity levels in the presence of rainfall events. The monitoring of this parameter in the water to be treated is, therefore, very important since water characterized by turbidity rates over 10 NTU in usually not treated; when this condition occurs, mainly during rainfall events, water from Bisagno river is not used for drinking water purposes. On the other hand, in the presence of lower turbidity values, in order to better manage the treatment train related to the settling of suspended solids, it is important to obtain an optimal dosage of the flocculant with reference to the turbidity rate. Turbidity, together with other parameters, is subject to continuous monitoring for the purpose. Another parameter subject to this kind of monitoring is residual chlorine; this measurement is linked to the dosage of sodium hypochlorite in the treatment train connected to disinfection.

Secondary disinfection is operated through an online monitoring system of residual chlorine enabling the plant operators to follow the disinfection process by adjusting and optimizing the amount of reactant. Therefore, by the plant chemical, physical and process parameters are on-line monitored before water is treated and at the entry point of the water distribution network.

Table 1 lists the water quality control parameters currently being tested at this pilot site. Currently, the pilot site is installed with individual off-the-shelf third party analysers to contentiously monitor turbidity, pH, conductivity and chlorine levels.

## 5. Design and Architecture

In this section, a preliminary version of the WaterSpy device architecture is presented, driven by the system requirements and application scenarios specified.

### 5.1. Overall Hardware Architecture

**Figure 4:**
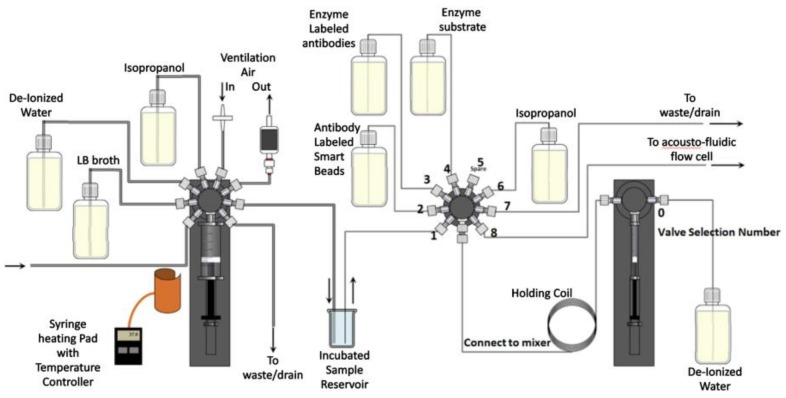
WATERSPY incubation module.

The WATERSPY device is composed of 5 main components, namely the TRITON device that provides the initial sample of 100 L (100 dm^3^), the MAF that concetrates and reduces the sample down to 1 mL (0.001 dm^3^), the incubation module (Figure 4), that automatically grows the target bacteria and enables a significant increase of sensitivity that is imperative due to the very low acceptable concentration of contaminants in the EU directives for water quality [30], and the detection component that includes an acousto-fluidic cell and the QCL-HOT photodetector detection system.

**Figure 5:**
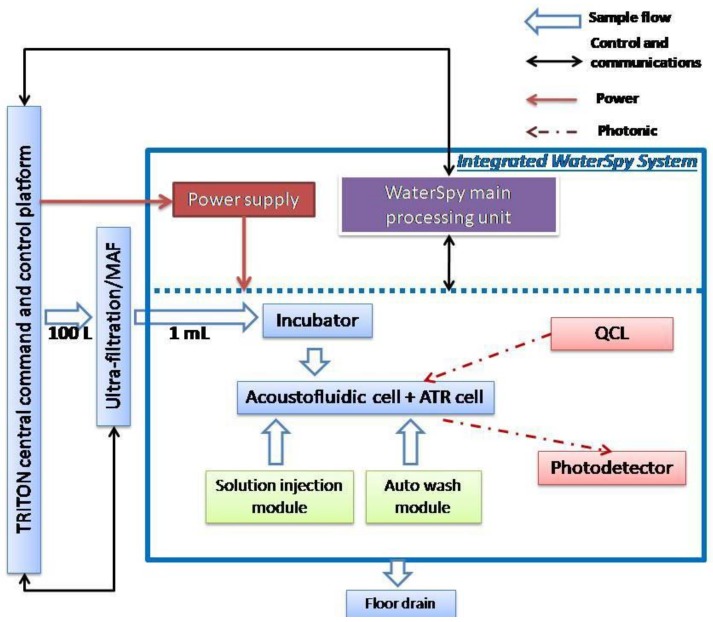
High level architecture configuration.

**Figure 6:**
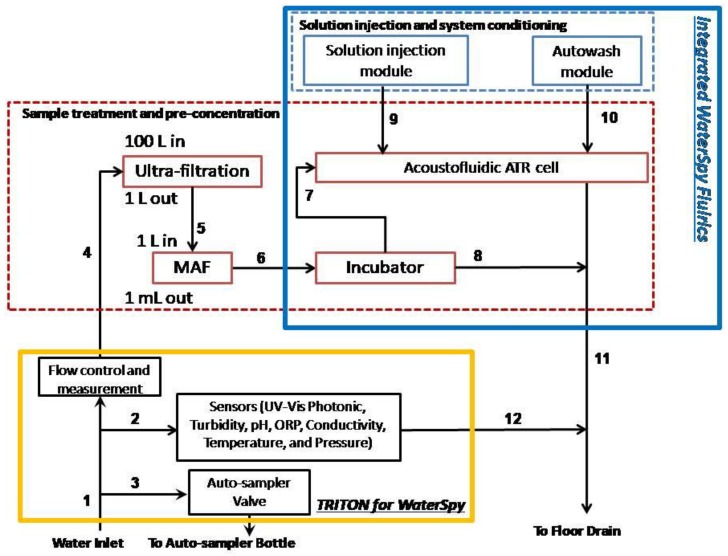
Integrated WaterSpy fluidic system.

An overall design of the system and its interactions can be viewed in Figure 5. As was mentioned earlier, the commercial water quality monitoring platform TRITON by AUG (Athens, Greece) is used to provide initial sample to the WaterSpy sampling treatment and pre-concentration system. The sample then is reduced down to 1 mL using the Ultra-filtration/MAF and is then fed in the Incubator that multiplies the bacterial population. After an incubation period of 4–6 h the incubated sample is redirected in an Acoustofluidic ATR cell, with “smart bead” carrying antibodies to retain the bacteria. After this process, enzyme-labelled antibodies are pumped in to interact with the retained bacteria to form an enzyme-labelled bacteria-bead conglomerate. Additionally, a substrate is injected in order to be converted to a specific product via the immobilized enzyme. Finally, the enzyme product is loaded and measured using the QCL and photodetectors. The integrated fluidic system can be viewed in Figure 6.

**Figure 7:**
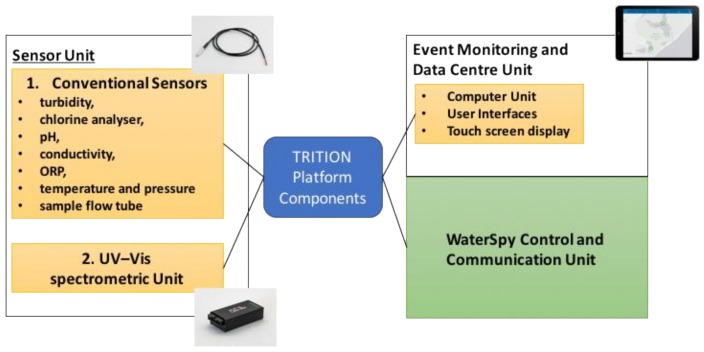
Configuration of WaterSpy based on TRITON platform.

Within the TRITON platform, three modular units will be included (Figure 7):

● Conventional sensor unit, including turbidity, free chlorine analyser, pH, conductivity, ORP, temperature and pressure sensors and sample flow tube;
● Spectrometric unit, including spectrometer, light source, optical flow cell, flow controller/transducer and autosampling valve;
● Event monitor and data centre unit (including computer unit and touch-screen display.)

### 5.2. Quantum Cascade Lasers

Quantum cascade lasers will be fabricated for the specific needs of WaterSpy project, as well as the driving electronics for them. Vernier QC lasers will be used in order to have a broader tuning range. However, as the difference in frequency is too high between the various target analytes, up to five different lasers will be fabricated. Following the first experiments conducted, the most prominent bands are at 1637 cm^−1^ (amide I), 1546 cm^−1^ (amide II), 1455 cm^−1^ (C-H deformation of CH_2_), 1399 cm^−1^ (C=O symmetric stretching of COO), 1233 cm^−1^ (P=O asymmetric stretching of PO_2_) and 1086 cm^−1^ (P=O symmetric stretching). Final selection will be done after further experimentation.

All beams need to be collimated and multiplexed. The simplest way is to send the beams to the target sequentially using a movable (i.e., Galvo) mirror. The overall size will depend on the type of packages. If the spectral gaps between the lasers are large enough (≥50 cm^−1^), then we will use dichroic mirrors which will significantly reduce the footprint but will also be more expensive and less flexible (cannot be used with other wavelengths). In both cases, the heatsink temperature will have to be stabilized and the heat from packages will need to be removed (due to Peltier cooler and laser).

### 5.3. Photodetector

**Figure 8:**
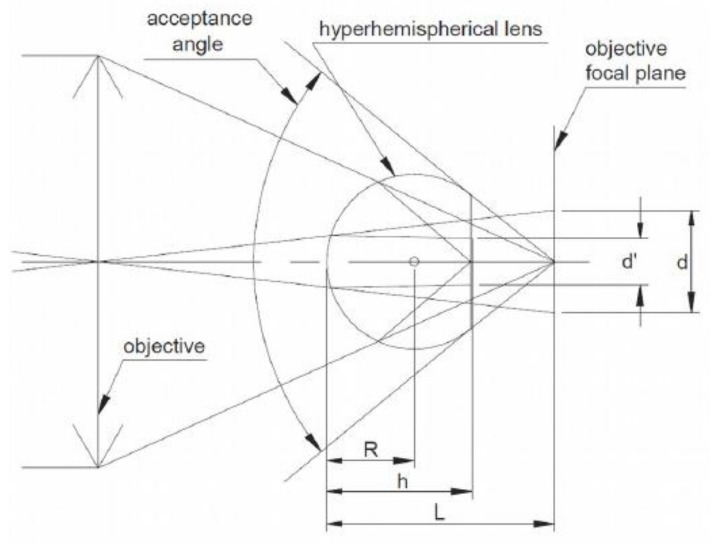
The GaAs hyperhemispherical lens.

A dedicated IR detector will be optimized for the needs of WaterSpy, and will be then integrated in a detection module consisting of: photodetector, preamplifier and TEC controller. The GaAs hyperhemispherical lens covering the active area will look as depicted in Figure 8, where d’ is the active detector size, d is the apparent detector size, R is the lens radius, h is the lens height. The acceptance angle of the hyperhemisphere made of GaAs is about 35°, although to some extent the manipulation of the field of view is also possible. The default package for the photodetector is the TO8 can.

### 5.4. Pre-Concentration and ATR Setup

#### 5.4.1. Pre-Concentration

The method for pre-concentration from a large volume (tens of m³) to some millilitres has been established by [31]. This method comprises three steps of concentrating particles (pathogens) in water:

(1) The first step includes filtering the water sample to remove large particles (>10 μm) but retain all the particles larger than 20 nm. The purpose of this first step is to concentrate the target and other particles from a large volume (even up to tens of m³) to 20 L volume (volumes can be adapted).
(2) The second step utilizes selective absorption of bacteria based on their material properties using monolithographic columns, actually resembling the HPLC (high performance liquid chromatography) method. Monolithographic columns serve as a pre-selection method, retaining only bacteria, opposed to other pre-concentrated organic and non-organic particles. These monolithic columns typically are made of epoxy based macroporous monolith. The bacteria are then eluted from the columns in black-flush, yielding a final volume of a few millilitres. This pre-concentrated bacteria suspension will be used as an input to the “ultrasound QCL-ATR device”.
(3) The third step is based on acoustic particle manipulation and acoustofluidics, a process guided by [32,33].

**Figure 9:**
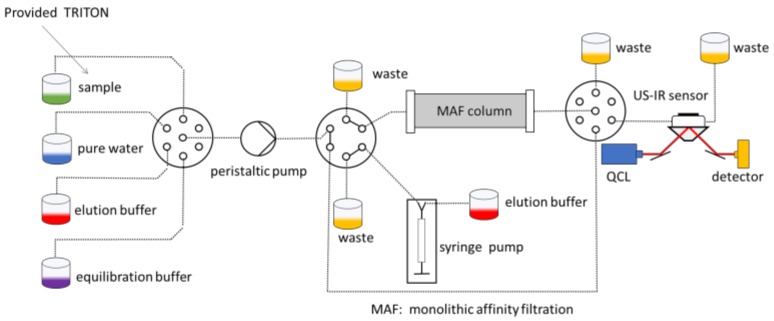
Details of the sample handling and pre-concentration system.

The geometry and operation of the ultrasound particle manipulation will be optimized so that the pre-concentrated bacteria can be reproducibly be directed to the “hot” spot of the ATR configuration, that is the position where total internal reflection of the laser beam takes place. The sample handling and pre-concentration approach is schematically presented in Figure 9.

#### 5.4.2. ATR Surface

**Figure 10:**
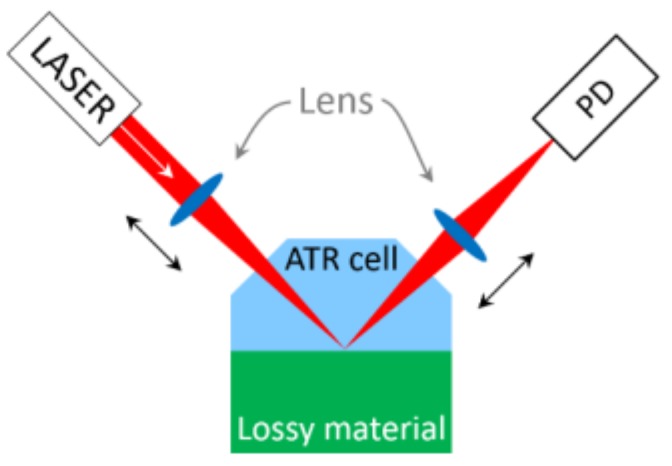
ATR setup.

To increase the probability to detect a bacterium on the ATR surface, the size of the laser spot on the ATR surface has to be considered. On one hand, the laser spot should be large to increase the probability of collecting bacteria on the surface, on the other hand the spot size should be small to increase the effect of a single bacterium or a few bacteria respectively on the sensor signal. To get a maximum amount of flexibility, we propose a setup as shown in Figure 10.

The first of the two additional lenses (right after the laser) allows adjusting the spot size on the ATR surface. The second lens (before the detector) focuses the divergent beam on the detector. The second lens brings the additional advantage, that the detector area may be smaller than without any lens. This is an advantage in terms of detector noise.

### 5.5. Processing Unit Architecture

**Figure 11:**
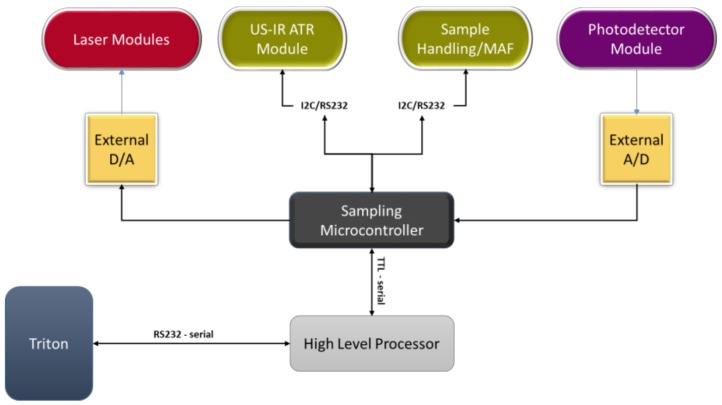
Two levels architecture.

**Figure 12:**
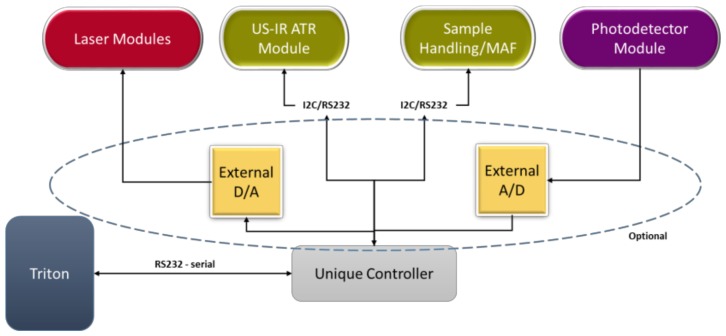
Single level architecture.

Two possible architectures have been designed for main processing unit configuration, a two-level (Figure 11) and a single-level one (Figure 12). The selection between them will be based on experimentation.

*Architecture I—Two level architecture*

In this approach, a low-level microcontroller (sampling microcontroller) is used to control the laser module and acquire the photodetector module’s output signal. The microcontroller also controls the timing of the laser and photodetector operation. Since the A/D and D/A units integrated on the available microcontrollers are not adequate for WaterSpy purposes (resolution and noise characteristics), external, dedicated A/D converters will be used.

Signal processing is done by the higher-level processor, which can be either a single-board computer or a compact controller. All signal processing algorithms will run on this processor. The high-level processor is also responsible for the communication with the TRITON. It is connected to the sampling microcontroller through a serial port.

The advantage of this configuration is that the most critical and demanding operations (sampling and controlling of lasers and photodetector) are decoupled from the signal processing tasks. In this way, we are ensuring that signals are acquired in the highest possible quality, without adding unnecessary noise to the measurement. The use of the external A/D and D/A units also increases the accuracy of the measurements.

The drawback is that the overall complexity of the architecture is higher with respect to solution II. It will also require the development of low-level firmware, which is less flexible to change and more difficult to debug. This can be a limitation, given the research nature of the project.

*Architecture II—Single level architecture*

In this configuration, all operations are controlled by a single controller. This, obviously, has to be a high-end controller or a computer that uses dedicated, hi-end, signal acquisition and analogue interfacing hardware. The external A/D and D/As, as in the previous approach, could also be used, to improve the converters’ characteristics, if the Unique controller does not offer adequate quality.

The advantage of this approach is the use of less hardware, thus reducing any hardware-related risks of failure. Additionally, it is simpler to debug and reconfigure, which is particularly useful during the testing phase.

The drawback is that the unique controller has to be a really hi-end device, which will probably increase the cost of the overall system. Additionally, the controller will have to be selected very carefully, since it will have to manage some very demanding tasks.

### 5.6. Firmware, Communications Architecture and Spectroscopy Optimization Approach

**Figure 13:**
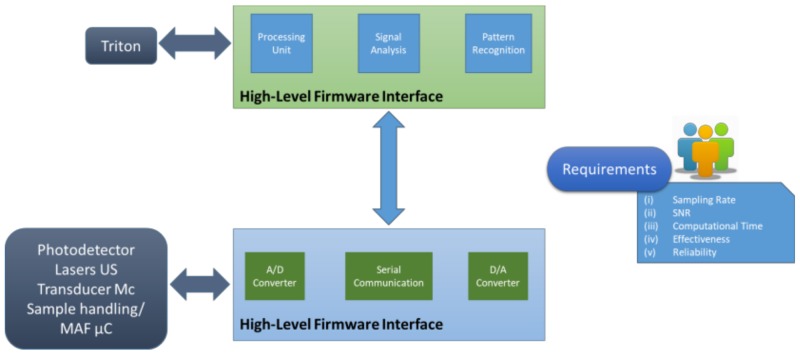
Firmware architecture main approach.

Figure 13 presents the firmware approach. As earlier explained, low-level and high-level firmware may co-exist in the same processor, depending on the architecture finally selected. By modulating the laser current and thus the wavelength, the spectrum of the sample can be measured directly. By applying the lock-in principle (synchronous detection), the bandwidth and the centre frequency of the measurement can be set arbitrarily. This significantly increases the signal to noise ratio. By adjusting the sampling frequency of the detection with respect to the modulation frequency, it is also possible to measure the first and second derivative of the spectrum directly. This is helpful to distinguish between different bacteria strains. Also, here the advantages of the synchronous detection apply.

It is important that the demands on the timing for this measurement principle are strictly satisfied. This has to be achieved independently of the architecture used (two-levels or single-level, as described above).

## 6. Preliminary Experimental Results

**Figure 14:**
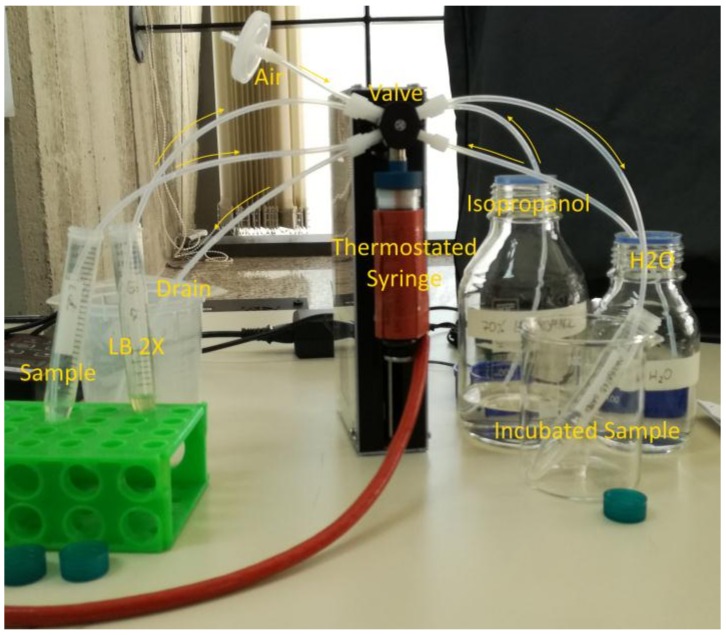
Configuration of the thermostated syringe.

In this section, we present some preliminary experimental results of the WaterSpy device components. In particular, we performed tests on the techniques that are incorporated in the device to test the bacteria incubation, a critical step in increasing the number of bacteria present in the sample to be identified by the QCL-photodetector. The thermostated syringe was tested and the amount of bacterial cells generated was compared with the product of a growth performed in a bacteriological incubator under the same conditions of temperature (37 °C) and time (6 h). Both bacterial growths (syringe and incubator) were performed without shaking. The thermostated syringe configuration is presented in Figure 14. 

The experiment was executed with 1 mL sample containing ~1000 bacterial cells re-suspended in deionized H_2_O, 1 mL LB 2X and 23 mL air, which were successively drawn into the 25 mL syringe and incubated at 37 °C. After 6 h the incubated sample was pushed out, centrifuged and the pellet washed and re-suspended in 250 l deionized H_2_O. Finally, the bacteria were counted in a Thoma chamber.

**Figure 15:**
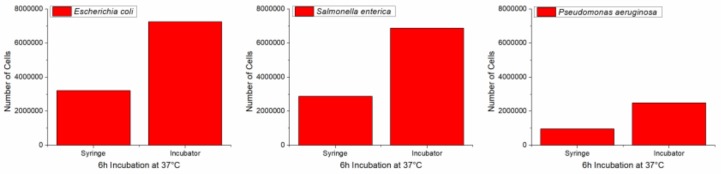
Results of the incubations in the thermostated syringe compared to respective bacteria incubations in the bacteriological incubator.

The results of the incubations in the thermostated syringe were compared with those of the respective bacteria incubations in the bacteriological incubator. From Figure 15, it is evident that all species underwent a growth rate decrease when incubated into the thermostated syringe that can be quantified as about 50% of the bacteriological incubator growth.

Moreover, enzyme-linked immunosorbent assay (ELISA) tests [34] were performed to assess whether the device can detect the presence of 1000 *E. coli* cells. In the smart beads configuration, the approach is based on a similar ELISA, but the enzymatic reaction product is detected by IR spectroscopy instead of visible. If in the smart beads approach it were possible to achieve a similar sensitivity of visible absorbance assay, the incubation step might possibly not be necessary. This has been a popular technique for control and measurement of proteins in water samples as in [35].

**Figure 16:**
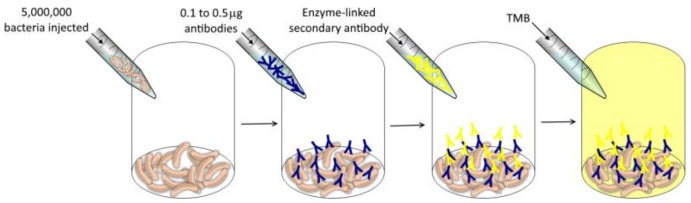
Previous ELISA experiments.

In the previous ELISA tests performed to characterize the antibodies, a big number of bacteria was used to entirely coat the bottom of the wells (~5 million bacteria), as the goal of the experiments was to study the affinity and the selectivity of the antibodies and if they are active in LB medium solution (see Figure 16).

**Figure 17:**
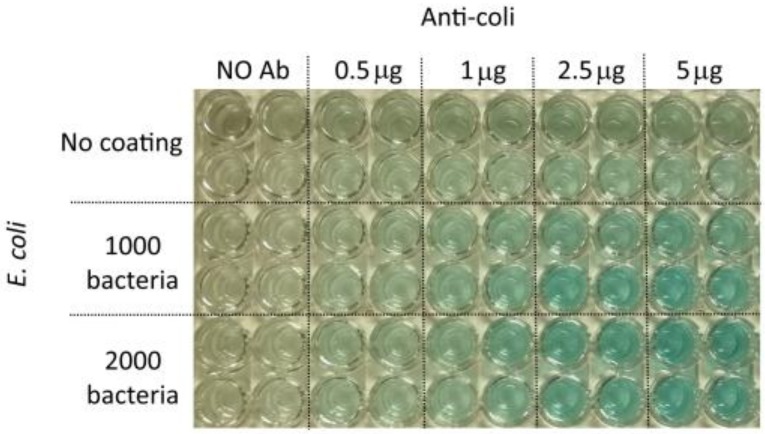
The microplate used for the experiment.

**Figure 18:**
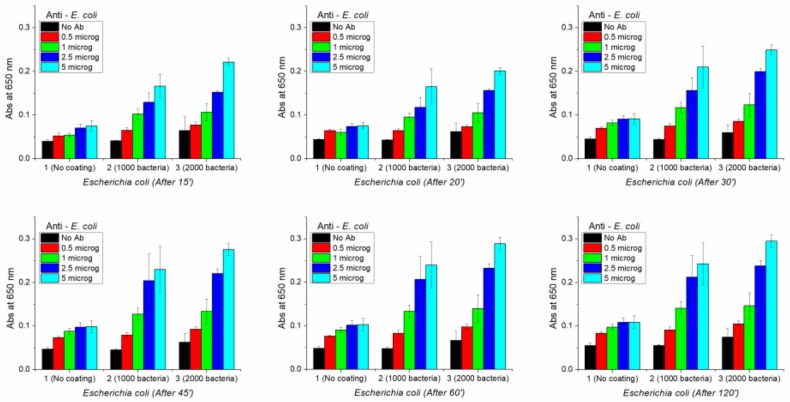
Experimental Results of the ELISA tests.

In order to check if the same ELISA assay can detect the presence of a very low number of bacteria, 1000 and 2000 *E. coli* cells were used to coat the bottom of the wells. In this experiment (Figure 17), different amounts of anti-*E. coli* were tested: 0.5, 1, 2.5 and 5 μg. After adding TMB, the absorbance was measured at 650 nm, without stopping the reaction with HCl. Each antibody/bacteria combination was repeated in four wells, and mean values and standard deviations were calculated. Absorbance was measured after 15′, 20′, 30′, 45′, 60′ and 120′. The results are reported below in Figure 18.

Despite very little a-specific binding of anti-*E. coli*, that can be observed by the very small increase of the absorbance in the no coated wells in the presence of increasing amount of antibody, this ELISA test indicates that it can detect the presence of a low number of bacteria. In particular, by using 5 μg of antibodies about 2.5-fold and 3-fold of the no-coating absorbance intensity were measured for 1000 and 2000 bacteria, respectively. These relative values were observed at all the times measured. The biggest absorbance absolute values both for 1000 and 2000 bacteria was observed after 120’, anyway after 60’ the absorbance stopped to increase significantly.

## 7. Conclusions

In this paper, we introduce the rationale and preliminary architecture and design of WaterSpy, a highly sensitive, portable photonic device for pervasive water quality analysis. The WaterSpy approach combines the use of advanced Quantum Cascade Lasers (QCLs) employing the Vernier effect, used as light source, as well as novel, fibre-coupled, fast and sensitive Higher Operation Temperature (HOT) photodetectors, used as sensors. Along with the new components, optimised laser driving and detector electronics, laser modulation, signal conditioning and signal processing techniques will be incorporated. Signal pattern analysis and machine learning techniques [36], [37] will also possibly be considered for possible further improvement of the performance. The approach builds on a set of solid principles including: cost-effectiveness, modularity, leveraging existing technologies and know-how, open software use maximization, high degrees of compatibility with existing technologies as well as with related European regulations. Preliminary results indicate that the designed device can effectively detect the presence of three pathogens with similar disinfection/inactivation procedures. The WaterSpy device will be installed by the drinking water portabilization plant located in Prato, Italy, operated by IRETI and included into the hydraulic infrastructures of the water supply system serving the city of Genoa.

## Figures and Tables

**Table 1:** The parameters currently being monitored at the pilot site in Prato, Italy.

Some Treatment Plants Inlet/Outlet Water Determinants	
Turbidity	Residual Chlorine
Temperature	Conductivity
pH	Organohalogen compounds
Iron, Cu, Ni	Colony count 37 °C
Manganese	Chlorites (plants with Chlorine Dioxide)
Aluminium	Chlorates (plants with Chlorine Dioxide)
COD	Total and Faecal Coliforms
Algae	Entero bacteria (plants fed with surface water)
*Escherichia coli*	Clostridium perfringens
